# Direct Metal Laser Sintering of the Ti6Al4V Alloy from a Powder Blend

**DOI:** 10.3390/ma15228193

**Published:** 2022-11-18

**Authors:** Lekhetho Ambition Ramosena, Thywill Cephas Dzogbewu, Willie du Preez

**Affiliations:** 1 Department of Mechanical and Mechatronics Engineering, Faculty of Engineering, Built Environment and Information Technology, Central University of Technology Free State, Bloemfontein 9301, South Africa; 2 Centre for Rapid Prototyping and Manufacturing, Faculty of Engineering, Built Environment and Information Technology, Central University of Technology Free State, Bloemfontein 9301, South Africa

**Keywords:** additive manufacturing, in situ, master alloy, pre-alloyed powder, single track

## Abstract

Additively manufactured Ti6Al4V parts have only seen major application in industries such as the aerospace and medical industries, mainly due to the high cost of production of the feedstock powder. In this article, the feasibility of in situ alloying a powder blend of elemental Ti and an Al–V master alloy to produce the Ti6Al4V alloy through direct metal laser sintering is presented and discussed. In a previous study, single track formation from this powder blend was studied and analyzed to determine the optimum principal process parameters suitable for this powder blend. These process parameters were employed in this study to produce single and double layers where the effects of varied hatch distance and the employment of a rescanning strategy on the surface morphology and alloy homogeneity were investigated. Lastly, in the current study, three-dimensional specimens were produced and analyzed to determine the alloy microstructure, homogeneity, part porosity and mechanical properties. The analyses revealed that a Ti6Al4V alloy with a density of up to 99.9% and corresponding tensile strength and ductility values of up to 942.9 MPa and 17% was produced. Furthermore, a minimum Al evaporation value of 7.2% was recorded. Therefore, in situ alloying can indeed be employed to produce high-quality Ti6Al4V parts from an elemental Ti and an Al–V master alloy powder blend.

## 1. Introduction

Additive manufacturing (AM) has been proven as a manufacturing technique capable of producing complex geometry components while ensuring minimal raw-material wastage as compared to conventional subtractive manufacturing techniques, such as turning and milling [1,2]. AM machine technologies have been grouped and categorized according to the type of feedstock material and energy source used [3]. Laser powder bed fusion (LPBF) is one of these categories, and it includes the direct metal laser sintering (DMLS) process, among others [3,4]. By virtue of its layer-wise production, the DMLS process can produce complex geometry parts that require little to no post-processing. However, the benefits offered by the DMLS process (and other AM processes) have only recently been realized by certain commercial industries that can afford the costly machines and materials employed in AM. To broaden the application of this technology, more affordable AM machines and materials must be developed and commercialized. This, therefore, warrants the need for the investigation and development of new and more cost-effective AM materials and methods to broaden the range of materials that can be used in AM technologies.

The Ti6Al4V alloy is the most common and widely used alloy of titanium. It has a combination of high strength, light weight, formability and corrosion resistance, which make it a desirable material for applications in many industries [5]. The light weight and high strength of this alloy make it appealing to the aerospace industry by virtue of its ability to adequately support aircraft components while adding less weight to the overall weight of the aircraft [6]. Furthermore, this alloy has received extensive amounts of attention from the biomedical industry due to its excellent biocompatibility. Several studies have been conducted where the feasibility of employing the Ti6Al4V alloy as a biomedical implant material has been demonstrated [7,8]. To date, Ti6Al4V parts have been produced with the DMLS process from pre-alloyed (PA) powder feedstock. In PA Ti6Al4V powder feedstock, each individual powder particle is an alloy of Ti6Al4V; therefore, the parts produced from this powder feedstock possess chemical and mechanical properties, which are comparable to those of the wrought Ti6Al4V alloy. The production of these PA powders is, however, costly, as it involves multiple thermo-mechanical processes, resulting in an expensive feedstock material. Consequently, the Ti6Al4V alloy is mostly used for high-value applications in industries where its good properties and characteristics justify its high price [9]. Therefore, the current research seeks an alternative approach by in situ alloying Ti6Al4V parts through the DMLS process.

The in situ alloying of a low-cost Ti6Al4V alloy from a mechanically mixed powder blend consisting of purely elemental powders has been investigated by several authors [10,11,12]. Simonelli et al. [10] investigated the appropriate preparation of an elemental powder blend suitable to be employed as feedstock for in situ alloying of the Ti6Al4V alloy. Different powder blending and preparation methods were investigated, and the microstructure and microhardness of the alloy produced from these powder blends were compared to the properties of the alloy produced from PA powder. This study provides a thorough understanding of the appropriate preparation of a low-cost elemental Ti6Al4V powder blend intended for in situ alloying. Another study by Polozov et al. [11] was focused on the effect of volume energy density (VED) on the alloy density, concentration and microstructure of the Ti6Al4V alloy produced from an elemental powder blend. The effect of the VED on the produced alloy was analyzed, and a Ti6Al4V alloy density above 99% was reported. Dong et al. [12] employed a powder blend consisting of hydrogenated-dehydrogenated Ti (HDH-Ti), Al and V powders to produce a cost-effective Ti6Al4V alloy through in situ alloying. A high-quality Ti6Al4V alloy with good mechanical properties was reported.

The literature demonstrates that in situ alloying can indeed be employed in AM processes to produce a high-quality, cost-effective Ti6Al4V alloy. However, issues such as alloy inhomogeneity and evaporation of the Al and V elements have been reported. Simonelli et al. [10] stated that the inhomogeneity reported in the study was caused by the inhomogeneity of the starting feedstock and emphasized that it is important to prepare feedstocks with controlled compositions, such as those offered by the PA powders. The in situ alloyed Ti6Al4V alloy produced by Polozov et al. [11] experienced an evaporation of the Al element, while the alloy produced by Dong et al. [12] experienced an evaporation of the Al and V elements. For this reason, Dong et al. suggested that a powder blend consisting of elemental Ti and 60Al–40V master alloy (MA) powders be employed to inhibit the evaporation of the alloying elements [12]. The Ti and 60Al-40V MA powder blend may result in a more controlled feedstock composition as compared to the purely elemental powder blend, therefore potentially resulting in a more homogenous Ti6Al4V alloy. The melting temperature of the Al–V MA ranges between 1650 and 1725 °C [13]; therefore, it can potentially inhibit the Al evaporation by virtue of the higher melting point of the alloy. A gap thus exists in literature for the investigation and production of the Ti6Al4V alloy through in situ alloying a Ti–60Al–40V MA powder blend, thereby determining the achieved homogeneity, composition and feasibility of this production approach. 

For a successful DMLS production of defect-free parts, it is mandatory that each individual powder should have its own set of material-related tailored process parameters. Yadroitsev et al. [14] stated that single tracks were essentially the building blocks of DMLS-produced parts; therefore, single track formation is the most important feature to investigate when dealing with a new powder material. Consequently, single track formation from a powder blend consisting of elemental Ti and 60Al–40V MA powders was investigated in a previous study [15]. A wide range of principal process parameters were used to deposit single tracks on a Ti6Al4V substrate using a layer thickness of 60 µm. Laser powers of 100, 150, 170 and 200 W and scanning speeds ranging from 0.4 to 1.8 m/s were employed. These tracks were analyzed to identify the most suitable principal process parameters for the deposition of optimum single tracks from the Ti-MA powder blend. The process parameters determined were laser powers of 100 and 200 W at scanning speeds of 0.6 and 1.2 m/s, respectively, using a layer thickness of 60 µm. In the current study, these principal process parameters were used to produce single and double layers, based on an optimum hatch distance suitable to produce good-quality single and double layers. Subsequently, the process parameters identified from the single track and layer production experiments were used to produce 3D parts from the powder blend. These 3D parts were characterized, and their properties were compared to those obtained with the PA powder. 

The purpose of this study is thus to investigate the feasibility of in situ alloying a powder blend consisting of elemental Ti and Al–V MA powders. The microstructure, chemical composition, mechanical properties and density of the produced alloy will be analyzed and the results presented. Upon the conclusion of this study, the process parameter sets that are suitable for in situ production of the Ti6Al4V alloy from the Ti-MA powder blend will be recommended.

## 2. Materials and Methods

### 2.1. Materials

**Figure 1:**
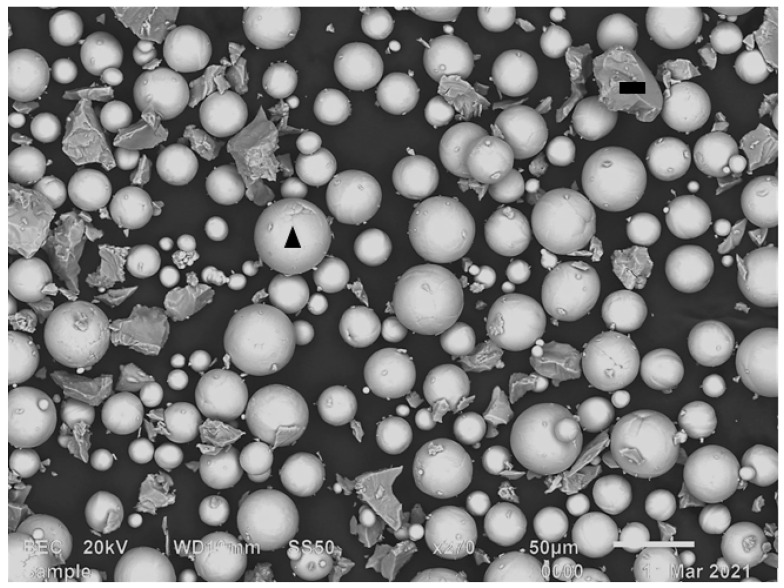
A SEM backscattered electron micrograph of the Ti-MA powder blend. The particle marked with the black triangle represents the Ti powder, while the particle marked with the black rectangle represents the MA powder particles.

A commercially pure (CP Grade 1) Ti (AP&C, Boisbriand, QC, Canada) and a 60Al–40V MA (Reading Alloys, Robesonia, PA, USA) powder blend (hereafter referred to as Ti-MA) were used in the study. Figure 1 shows a scanning electron microscope (SEM) micrograph of a typical sample of the Ti-MA powder blend. The spherical CP Ti powder was argon gas atomized, while the irregularly shaped MA powder was produced by an aluminothermic smelting process followed by ball milling. The particle size of the spherical Ti powder was in the range of 20–45 µm, and the irregularly shaped MA powder had a particle size ranging from 20 to 80 µm.

Inductively coupled plasma–optical emission spectroscopy (ICP-OES) analyses using the Spectro Acros FHE12 (Spectro AMETEK Inc., Kleve, Germany) were conducted on the powders to determine the purity and the elemental composition of the feedstock powders (see Table 1). The analyses revealed that the Al–V MA had an Al concentration of 54.8 wt.%, which was below the target concentration of 60 wt.% required for the production of the Ti6Al4V alloy. The concentration of the V within the MA powder was above the required final concentration of 40 wt.%. Despite the concentration of the Al in the MA powder being below the target composition, the overall chemical composition of the powder feedstock shown in Table 1 was expected to be adequate to ensure a post-production chemical composition, which would be within the range specified in the ASTM F2924-14 standard [16].

### 2.2. Sample Production and Analyses

#### 2.2.1. Single and Double Layers

An EOSINT M280 (Electro Optical Systems GmbH, Krailling, Germany) machine from the Centre for Rapid Prototyping and Manufacturing at the Central University of Technology, Free State, was used to produce the single tracks, single and double layers, and 3D parts. Two principal process parameter sets, namely, laser powers of 100 and 200 W with corresponding scanning speeds of 0.6 m/s and 1.2 m/s, respectively, were used to produce single and double layers from a powder layer thickness of 60 µm. Hatch distances of 80 µm, 90 µm and 100 µm were used for producing the layers to investigate the effect of track shifting and identify the optimum hatch distance suitable for the layer production. Three trial samples were produced at each hatch distance of 80 µm, 90 µm and 100 µm for each of the two principal process parameter sets. 

A rescanning strategy was employed on the produced layers to investigate the effect of this strategy on layer morphology. A Surftest SJ-210 (Mitutoyo, Aurora, CO, USA) portable surface roughness testing machine was used to analyze the roughness of the layers before and after the rescanning strategy. A JiangSu Fangzheng (Taizhou, Jiangsu, China) electrical discharge machine (EDM) was used to cross-section the samples in preparation for the cross-sectional analyses needed to determine the bonding properties of the super-positioned single tracks at the different hatch distances. 

Optical imaging of the single and double layers was conducted using a Zeiss-Axio Scope.A1 optical microscope (ZEISS, Oberkochen, Germany). Secondary electron imaging (SEI), backscattered electron imaging (BEI) and energy dispersive X-ray spectroscopy (EDS) analyses were conducted on the specimens in a high-resolution JEOL (Tokyo, Japan) JSM-7800F scanning electron microscope (SEM). The SEI mode was used to further analyze the surface and topology features of the produced layers, while the BEI mode and EDS were employed to investigate the elemental homogeneity of the produced layers.

#### 2.2.2. Three-Dimensional Specimens

Two optimal process parameter sets, each consisting of laser power, scanning speed, layer thickness and hatch distance, were obtained from the preceding analyses and employed to produce 3D specimens. Process parameter set 1 (PPS1) consisted of a laser power and scanning speed combination of 100 W, 0.6 m/s and a hatch distance of 80 µm using a layer thickness of 60 µm. Process parameter set 2 (PPS2) consisted of a laser power and scanning speed combination of 200 W, 1.2 m/s, a hatch distance of 80 µm and a layer thickness of 60 µm. 

**Figure 2:**
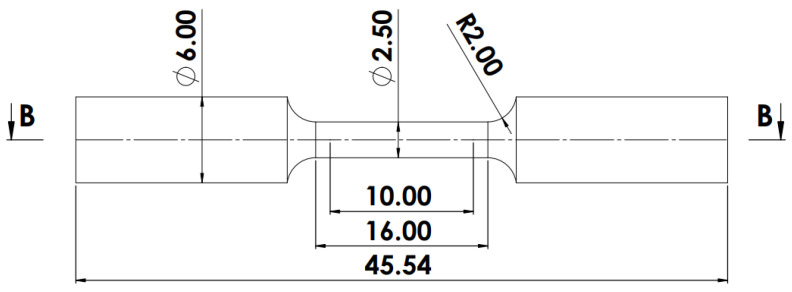
The dimensions of the scaled-down tensile test specimens, as specified in the ASTM E8/E8M standard. All dimensions are in mm.

For each of the two process parameter sets, three 3D cubes and four scaled-down tensile test specimens were produced. The cubes had dimensions of 8 × 7.5 × 7 mm (L × B × H), while the round tensile test specimens were scaled down and built in compliance with the ASTM E8/E8M–15a standard [10] according to the dimensions shown in Figure 2. The cubes were produced on one 6 mm thick Ti6Al4V substrate, while the tensile test specimens for each PPS were produced on a different Ti6Al4V substrate.

**Figure 3:**
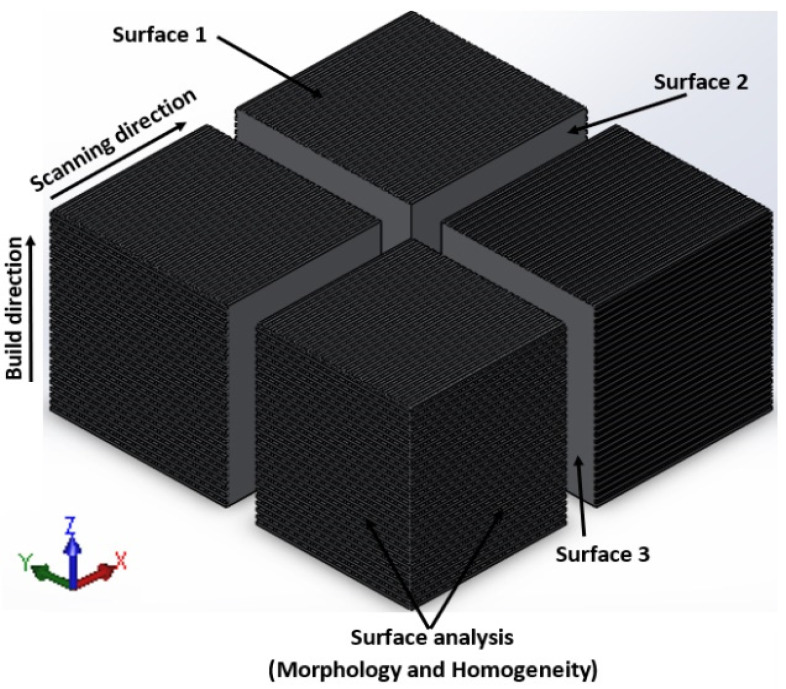
Orientation of the first cross-sectioned cube for each process parameter set.

The JiangSu Fangzheng EDM was used to cut the cubes from the substrate in their “as-built” (AB) condition. For each process parameter set, two of the three cubes were cross-sectioned into four equal pieces, as shown in Figure 3, using the EDM, while the last cube was kept as produced. The cubical pieces of the first cross-sectioned cube were mounted in resin using a Struers (Cleveland, OH, USA) CitoPress-15 hot mounting press for microstructural analyses, which were performed on the three surfaces shown in Figure 3. With reference to the coordinate system and surface labels shown in Figure 3, Surface 1 refers to the x-y plane (scanning plane), which is the top surface of the produced cube. Surface 2 refers to the x-z plane, while Surface 3 refers to the y-z plane. An analysis of the surface morphology was conducted using the JEOL JSM-7800F SEM on the surfaces that were parallel and perpendicular to the scanning direction shown in Figure 3. The second cross-sectioned cube was sent to the South African Nuclear Energy Corporation SOC Ltd. (NECSA) for ICP-OES compositional analysis, where all the cubical pieces were dissolved in acid and analyzed. The third AB cube was sent for computed tomography (CT) scanning at Stellenbosch University (SU) to determine the percentage porosity. The CT scanning facility at SU employs a General Electric (Boston, MA, USA) Phoenix Nanotom^®^ Nano CT system equipped with a 180 kV/15 W ultra-high-performance nano-focus X-ray tube.

The tensile test specimens were first stress relieved (SR) at 650 °C for a soaking period of 3 h and subsequently annealed at 940 °C for 2 h before they were cut from the substrate. The grip sections of the tensile test specimens were threaded, while the gauge length sections were machined using a Masteel (Vancouver, BC, Canada) LEADWELL T/7AM computer numerical control (CNC) TURN-MILL machine. Once the tensile tests had been conducted, the fracture surfaces were investigated in a high-resolution JEOL JSM-7800F SEM. Additionally, one fractured tensile test specimen was sectioned in half along section B-B shown in Figure 2 to have an indication of the obtained heat-treated (HT) microstructure.

## 3. Results and Discussion

### 3.1. Single and Double Layers

#### 3.1.1. Top and Cross-Sectional Surface Morphology

**Figure 4:**
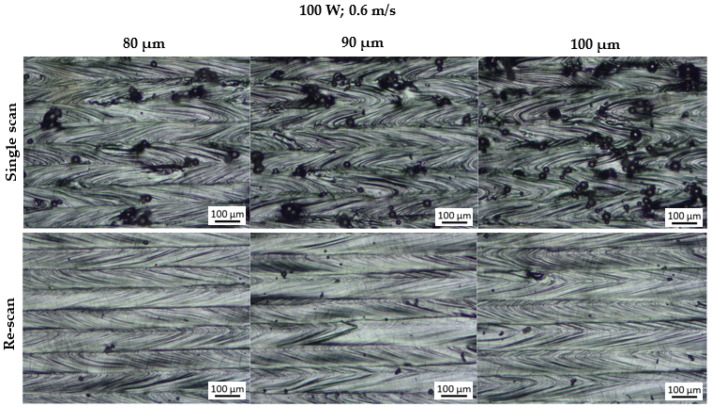
Optical micrographs of the top surface morphology of the double layers produced at 100 W; 0.6 m/s and hatch distances of 80, 90 and 100 μm.

**Figure 5:**
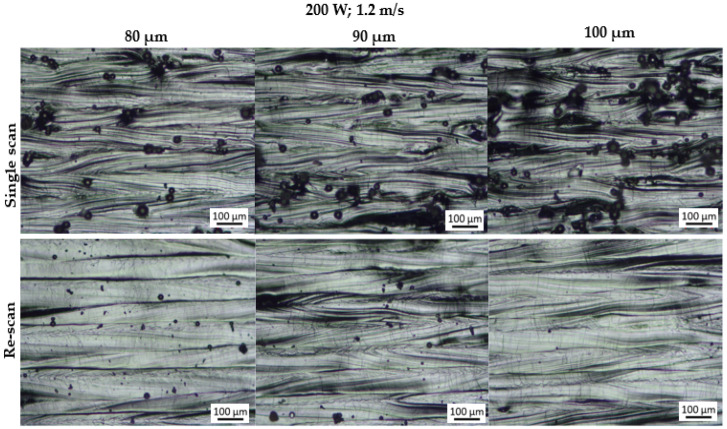
Optical micrographs of the top surface morphology of the double layers produced at 200 W; 1.2 m/s and hatch distances of 80, 90 and 100 μm.

Figure 4 and Figure 5 display the morphology of the produced layers at each hatch distance before and after the rescanning strategy (double scan). The single and double layers exhibited a similar kind of morphology; therefore, only the double layers are displayed for illustrative purposes.

**Figure 6:**
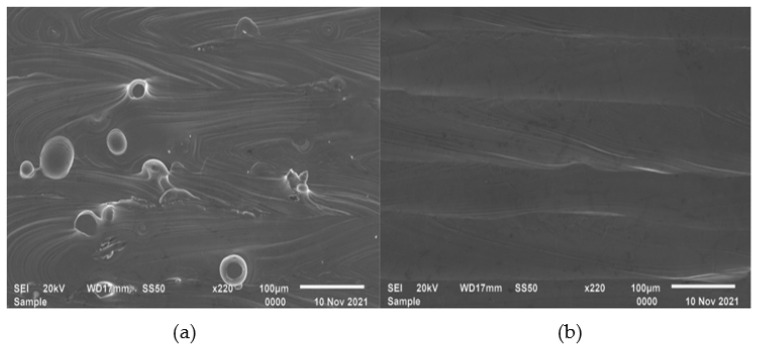
SEM SEI micrographs of double layers produced in PPS1 before (**a**) and after (**b**) rescanning.

The single track analysis revealed that the single tracks exhibited satellites formed on their edges [15], which in turn resulted in satellites forming on the surfaces of the single and double layers. This observation is in agreement with the findings of Yadroitsev et al. [14], where it was found that the surface morphology of the layers produced in LPBF depends on the characteristics of the single tracks. It was further observed that the layers produced at a hatch distance of 100 µm exhibited more satellites than those produced at a hatch distance of 80 µm. Similarly, the findings from other studies also suggested that the number of satellite particles formed on a layer increases with an increase in the hatch distance [17,18]. In a different study [19], these satellite particles had an adverse effect on the mechanical properties of the final 3D component. However, the rescanning strategy was effective in removing these satellite particles, ensuring a defect-free layer surface. Spatter particles are essentially imperfectly melted powder particles; hence, a second laser scan was able to completely melt and redistribute them into the layer. Yadroitsev et al. [14,20] explained that the mechanism of removing these particles was fueled by the difference between the thermo-physical conditions of the powder and the solid material. They suggested that the absorbance of the solid material was lower than that of the powder material; therefore, in the second laser scan, the imperfectly melted powders absorbed more laser radiation, thus leading to complete melting and redistribution into the bulk material [20]. This observation by Yadroitsev et al. was confirmed by the observations and results obtained in the current study. The effect of this strategy is also illustrated in the SEM micrographs in Figure 6 of the double layers before and after the rescanning strategy.

**Figure 7:**
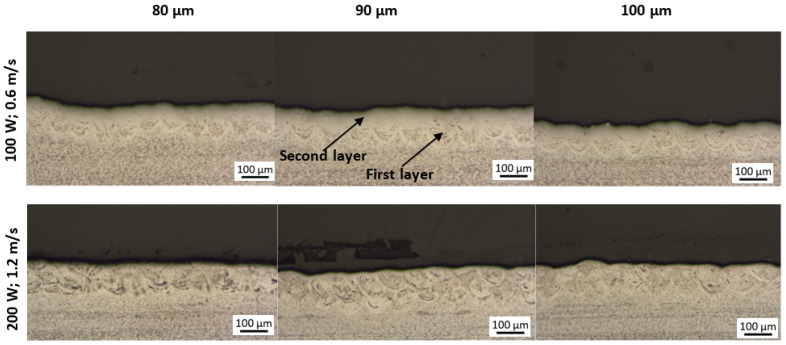
Cross-sectional views of double layers produced at 100 W; 0.6 m/s and 200 W; 1.2 m/s at hatch distances of 80, 90 and 100 µm.

Considering the average width of the tracks deposited from PPS1 and PPS2 (152 and 145 µm, respectively) [15], a hatch distance of 80 µm would allow a greater portion of the single tracks to overlap with each other as compared to hatch distances of 90 and 100 µm. The cross-sectional analyses of the double layers (Figure 7) indicate that the layers produced at a hatch distance of 80 µm did indeed have a better track-to-track overlapping (binding) as compared to those produced at 90 µm and 100 µm for both process parameter sets. It is worth mentioning that no gaps between the tracks and no delamination between the layers were observed throughout the range of hatch distances used for single and double layer production.

**Figure 8:**
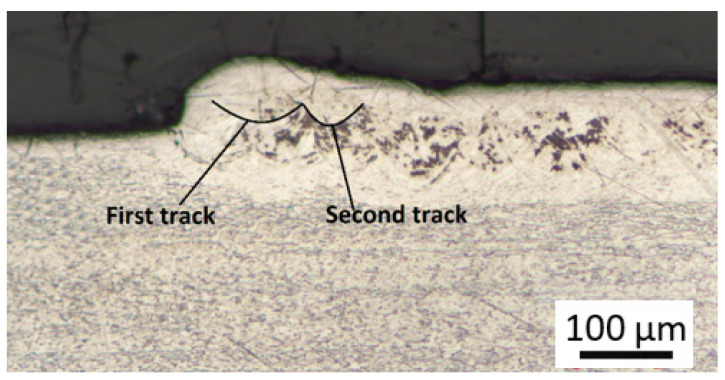
Denudation effect observed on a cross-section of the produced double layers.

The effect of substrate denudation was observed in the cross-sectional analyses of the single and double layers. From Figure 8, the first few tracks that formed the layer appear to be higher and wider than the tracks in the middle span of the layer. Similar studies [20,21] suggest that the deposition of the first track will result in a denudation zone on the substrate during layer production. When the second track is deposited using a hatch distance that is less than the width of the denudation zone, it will then appear to be lower than the first track due to the decrease in available powder. Additionally, the last track in the layer will also be higher than the middle tracks because there is an excess of powder remaining in the powder bed available for the formation of this track on the periphery of the laser spot. Ultimately, the denudation effect results in dimensional inaccuracy and defects such as chains of pores within the final 3D component; therefore, an allowance for the variation of the track height should be made during the production of 3D components [14,20,21]. However, in the current study, defects such as porosity, solidification cracks and layer delamination were not observed in the sectioned areas of the samples. The absence of these defects signifies an optimum combination of process parameters suitable for the DMLS production of 3D components [17].

#### 3.1.2. Surface Roughness

**Figure 9:**
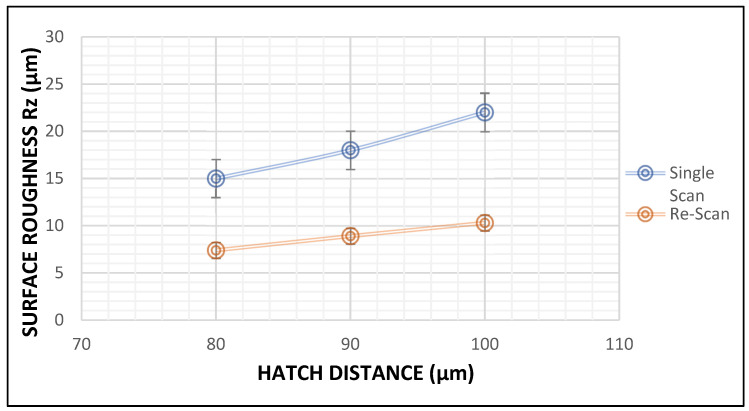
Plot of surface roughness (µm) vs. hatch distance (µm) for double layers produced at 200 W and 1.2 m/s.

The satellite particles observed in Figure 4 and Figure 5 are considered as peaks in surface roughness measurements. The value of Rz was used to characterize the surface roughness because it accurately describes the average distance between the peaks and valleys within the sampling length [22]. Since these peaks increased with an increase in the hatch distance, it was found that the surface roughness of the layers also increased with an increase in the hatch distance, as shown in Figure 9. The removal of the satellites formed on the surface of the layers during the rescan resulted in a reduction in the surface roughness of the layers, as seen in Figure 9. The value of Rz decreased by an average of 51% after the rescanning process. This decrease in surface roughness after rescanning was also reported by other authors [14,20].

#### 3.1.3. Layer Homogeneity

**Figure 10:**
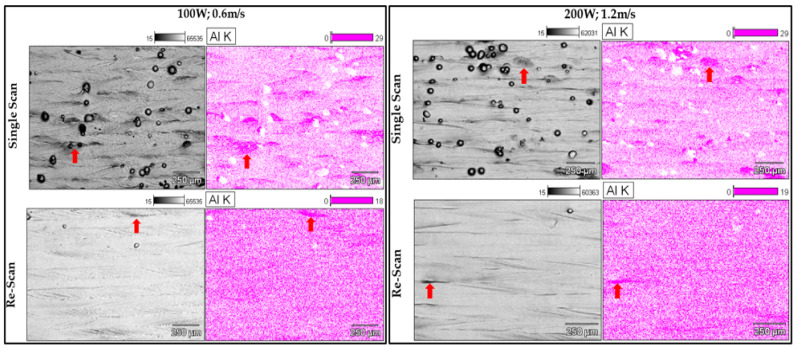
BEI images and Al K EDS maps of double layers at 100 W; 0.6 m/s and 200 W; 1.2 m/s for a hatch distance of 80 µm.

An indication of the degree of homogeneity of the in situ alloyed Ti6Al4V layers can be seen in the single and double scan micrographs in Figure 10. 

From the relative intensity scale (top right of the micrographs), a significant Al inhomogeneity exists mostly at the edges of the single tracks that formed the layer (indicated by red arrows). There are two factors that could contribute to this inhomogeneity. Firstly, Chan et al. [23] found that it could be due to the uneven distribution of energy by the laser beam, which created the melt pool from the metal powders. This uneven distribution, paired with the non-stationarity of the energy source, resulted in a non-uniform temperature in the melt pool when a single track was produced. The temperature of the melt pool would thus be higher at its center and lower at its edges [14]. Therefore, the degree of melting and mixing would be less thorough at the edge of the melt pool due to the relatively lower temperature. Additionally, the high solidification rate typical of the DMLS process promoted inhomogeneity by not allowing adequate time for diffusion before solidification [24]. The inhomogeneity could also be due to insufficient mixing of the powder blend before it was employed in the DMLS process. The MA powder had an irregular morphology (Figure 1), which could limit the degree of powder blending and lead to the consequent inhomogeneity of the powder blend. Simonelli et al. [10] stated that the inhomogeneity of the starting material would cause inhomogeneity in the final product, which could only be removed by post-sintering processes. As expected, the rescanning strategy did homogenize the alloy by melting and redistributing the satellites into the bulk of the alloy. Nevertheless, areas relatively higher in Al concentration (indicated by the red arrows) can still be seen in the elemental mapping micrographs in Figure 10. However, it should be possible to improve the homogeneity of the alloy by improving the homogeneity of the powder blend before it is employed in the DMLS process. This could be achieved by replacing the irregularly shaped MA powder with spherical MA powder, allowing more homogenous mixing of the employed powders.

### 3.2. Three-Dimensional Parts

#### 3.2.1. Microstructural Analysis

**Figure 11:**
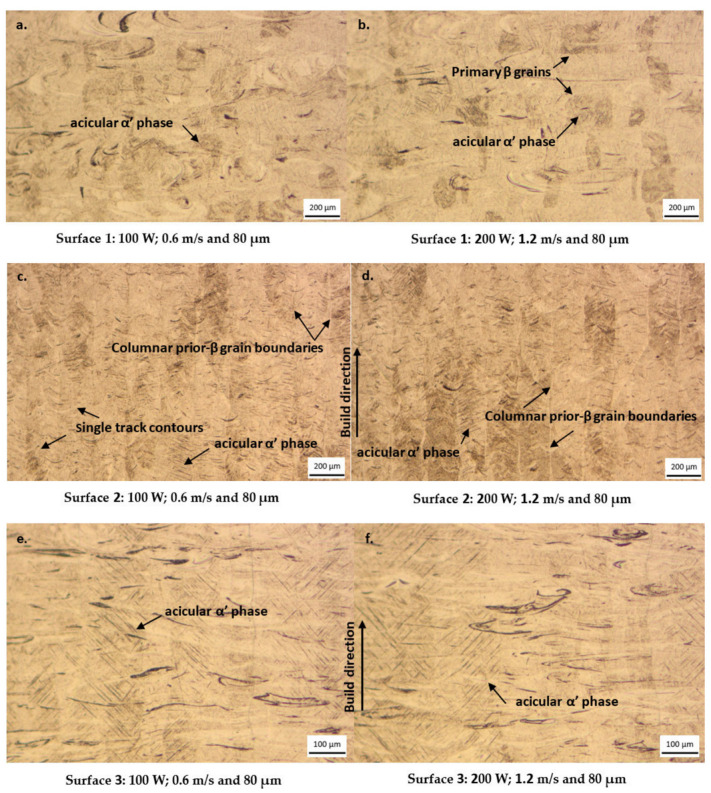
Microstructures of the cross-sectioned, polished and etched surfaces of the AB Ti6Al4V specimens. (**a**,**b**) Surface 1, x-y plane. (**c**,**d**) Surface 2, x-z plane, along the building direction. (**e**,**f**) Surface 3, y-z plane, along the building direction.

During the conventional DMLS production of the Ti6Al4V alloy, the PA metal powder is laser melted to form a melt pool, which then rapidly solidifies [25]. Rapid solidification is caused by the high cooling rate (up to 10^8^ K/s) typical of the DMLS process [26,27]. The resulting microstructure is a binary (α + β) microstructure, consisting of acicular martensitic α needles (laths) within columnar prior-β grains [27]. The microstructures obtained from the polished and etched AB specimens produced from the powder blend are shown in Figure 11.

The microstructures of the sectioned AB cubes generally consisted of a fine needle-like acicular martensitic α’ phase contained within columnar prior-β grains, typical of the additively manufactured Ti6Al4V alloy, as reported by other authors [10,26,28]. This martensitic microstructure is distinctive for DMLS-produced parts due to the rapid cooling and solidification rates found in this process [29,30]. The microstructure of Surface 1 (Figure 11a,b) consists of the acicular α’ phase within the primary β grains. Some single track contours can be seen in the microstructures shown in Figure 11c,d. From these micrographs, it is evident that the hatch distance employed to produce these specimens was optimum, as the u-shape single track contours overlap completely with each other. This observation is consistent with the one made in the double layer cross-sectional analysis shown in Figure 7. The columnar prior-β grains, which have grown in a direction parallel to the building direction, can be seen on these micrographs. Within these columnar prior-β grains, fine acicular martensitic α’ laths are visible. Like the micrographs of Surface 1 and Surface 2 of the cubes, fine acicular martensitic α’ laths within the prior-β grains could also be observed on the micrographs of Surface 3 (Figure 11e,f). No layer delamination or inter-layer pores could be identified from these micrographs. The micrographs shown in Figure 11 demonstrate the microstructural anisotropy found in the as-built Ti6Al4V parts. Since the mechanical properties of Ti6Al4V parts are highly dependent on the microstructure [31], this could result in some anisotropy in the mechanical properties of the built part.

**Figure 12:**
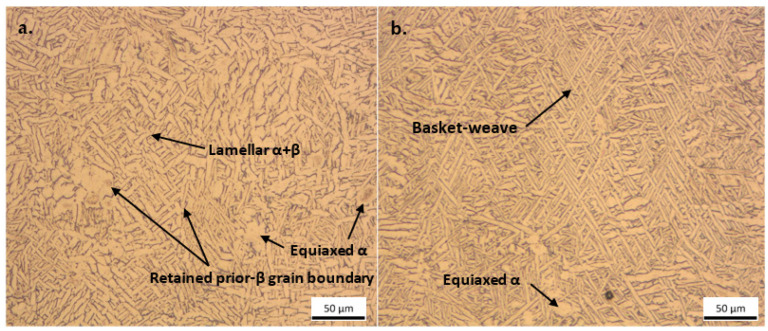
Microstructures obtained after stress relieving at 650 °C for 3 h and annealing at 940 °C for 2 h for (**a**) PPS 1 and (**b**) PPS 2.

The large temperature gradients that exist during the DMLS production of Ti6Al4V parts normally induce thermal residual stresses within the produced parts. These residual stresses can cause the built parts to warp or deform when they are removed from the building platform [32]. Therefore, the produced tensile test specimens were stress relieved (SR) at a temperature of 650 °C with a soaking period of 3 h. In this study, the SR treatment was effective in alleviating the internal thermal residual stresses, and no deformation or warping of the parts were observed when they were wire cut from the substrate. The annealing heat treatment performed at 940 °C for 2 h on the tensile test specimens caused the decomposition of the martensitic α’ phase into a more ductile microstructure, which generally consisted of a lamellar α + β phase (Figure 12) with retained prior-β grains.

#### 3.2.2. Surface Quality

**Figure 13:**
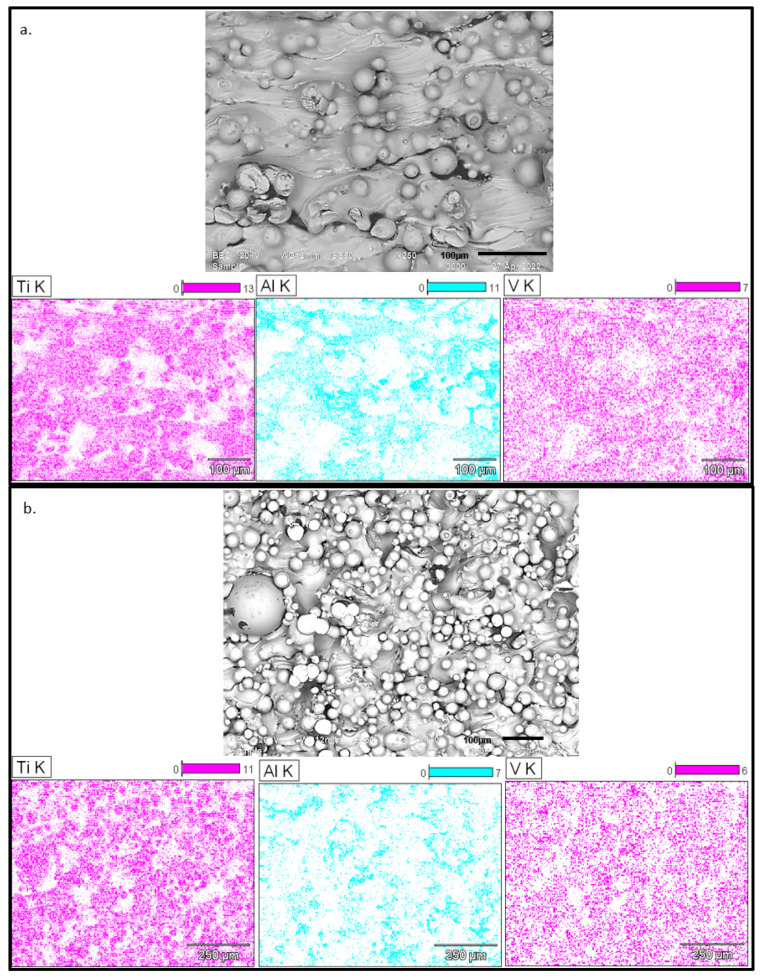
BEI image and Ti, Al and V K EDS maps of the morphology and homogeneity of the surfaces of AB cubes produced in (**a**) PPS1 and (**b**) PPS2.

Ideally, DMLS-produced parts should have a high surface quality and require minimum surface post-processing [26]. However, due to the intrinsic characteristics of the DMLS process, the parts produced usually exhibit varying surface quality with respect to surface homogeneity for in situ alloyed parts [33]. This variation in surface homogeneity is caused by the different constituents of the powder blend that agglomerate as imperfectly melted powders on the surfaces of the produced parts due to the excessive heat energy from the laser beam. The surface morphology and the variation in surface homogeneity of the produced parts can be seen in Figure 13a,b.

In a powder bed consisting of a PA feedstock, each individual powder particle is essentially already an alloy consisting of the composition, which is required in the final built part [9]. When these PA powders are imperfectly melted, they agglomerate on the surfaces of the part during layer-wise production. However, these imperfectly melted PA powders do not result in micro-inhomogeneity on the surface of the produced parts, as they bear the same composition as the bulk of the part being produced. This is, however, not the case for a powder blend intended for in situ alloying. The powders surrounding the part being built are not of the same composition as the bulk of the in situ alloyed part. Therefore, the excess heat energy from the melt pool may reach any of the randomly distributed constituents of the powder blend, which can agglomerate onto the surface of the part as imperfectly melted powders, resulting in micro-inhomogeneity on the surface of the part. An SEM-EDS analysis conducted on the AB surfaces of the cubes produced from PPS1 revealed that the imperfectly melted powders that agglomerated on the outer surfaces of the cubes are indeed the different constituents of the powder blend, as can be seen in Figure 13a,b. This observation thus warrants the need for surface post-processing on parts that were in situ alloyed in the DMLS process. Partially molten Ti powder particles can be clearly identified by their spherical morphology in the BEI images of the surfaces of the AB cubes (Figure 13a,b), while the MA powders cannot be directly identified, but a variation in contrast can be seen. This variation in contrast can be attributed to the inconsistent composition within the analyzed area. A Ti powder particle that has a particle size in the upper limit of the particle size range is visible in Figure 13b. The Ti K EDS map in Figure 13b confirmed that this powder particle was indeed a Ti powder particle. A great deal of surface inhomogeneity can be seen in the elemental EDS maps performed on the surfaces of the cubes, which is a clear indication of the inhomogeneity of the surfaces of the in situ alloyed parts.

#### 3.2.3. Chemical Composition

The chemical composition of the produced Ti6Al4V cubes is provided in Table 2. 

The major obstacle anticipated with the in situ alloying of the Ti6Al4V alloy was the evaporation of the Al during powder fusion due to its low melting temperature of 660.2 °C [11,12,13]. The use of the Al–V MA powder was intended to restrain the Al evaporation by virtue of the higher resultant Al–V alloy melting temperature as compared to the low melting temperature of the elemental Al powder. However, the results obtained from the ICP-OES analysis suggest that some evaporation of the Al powder did take place during production. As shown in Table 2, the measured Al concentrations were 4.73 and 5.11 wt.% for PPS 1 and PPS 2, respectively. Taking into consideration the actual concentration of the Al in the MA powder (54.8 wt.%, see Table 1), PPS1 and PPS2 yielded evaporation percentage values of 13.7% and 7.2%, respectively. The PPS 1 Al concentration was below that specified in the standard, while PPS 2 had an Al value within the tolerance specified in ASTM F2924–14. These values are, however, lower than the Al evaporation value (23,3%), which has been reported in a similar in situ study by Dong et al. [12]. 

The unexpected occurrence, however, was the decreased V content present in the AB Ti6Al4V alloy. With reference to the starting composition of the MA powder (see Table 1), it is evident that PPS1 and PPS2 yielded V evaporation values of 18.66% and 14.29%, respectively, which are similar to the V evaporation values (17.5%) reported by Dong et al. [12]. This decrease in V content can be attributed to the evaporation of the V_2_O_5_ compound that formed during sintering as a result of the reaction between the V and the O_2_, as has been presented by Dong et al. [12].

The oxygen and nitrogen concentrations were within the limits specified in the standard. Oxygen and nitrogen are interstitial elements in Ti6Al4V, and their concentration should always be controlled not to exceed 0.2 and 0.05%, respectively, because an excess in these elements would lead to an alloy with increased strength but decreased ductility [34]. These compositional results demonstrated that it was indeed possible to in situ alloy a Ti-MA powder blend to produce a part with the Ti6Al4V alloy composition. However, an appropriate concentration of the starting feedstock and the process parameter set employed would be required to produce the correct alloy.

#### 3.2.4. Porosity

**Figure 14:**
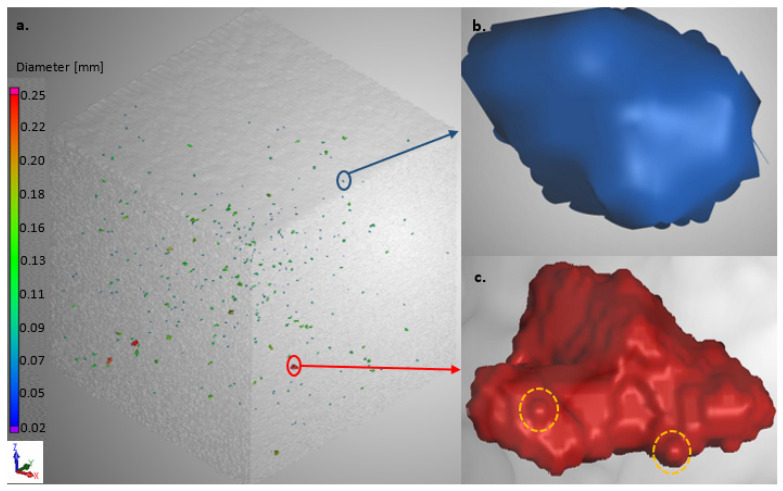
Micro CT scans of the AB Ti6Al4V cubes produced from PPS 1: (**a**) the overall porosity distribution within the cube, (**b**) gas pore, (**c**) lack of fusion pore exhibiting unmolten Ti morphology (circled areas). The color codes of the pores displayed in (**b**,**c**) correspond to the diameter scale shown in (**a**).

**Figure 15:**
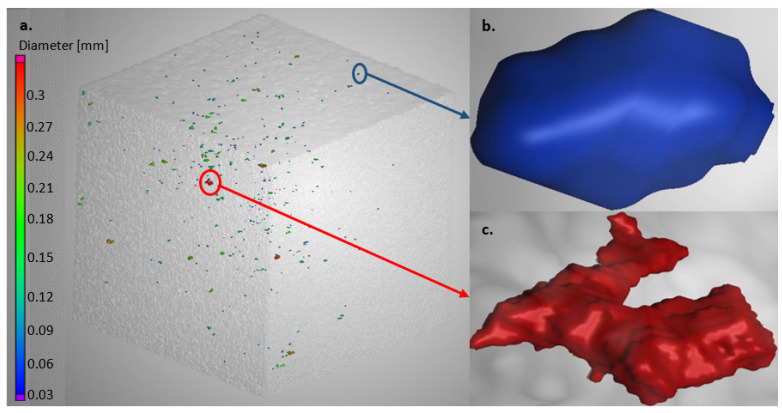
Micro CT scans of the AB Ti6Al4V cubes produced from PPS 2: (**a**) the overall porosity distribution within the cube, (**b**) gas pore, (**c**) lack of fusion pore. The color codes of the pores displayed in (**b**,**c**) correspond to the diameter scale shown in (**a**).

The results obtained from the CT scans of the 3D cubes produced with process parameters sets 1 and 2 are displayed in Figure 14 and Figure 15, respectively. 

DMLS-produced parts are usually affected by two types of pores: gas pores and lack of fusion pores [26,35]. Gas pores are usually randomly distributed in the produced part and are typically spherical with a diameter ranging between 1 and 100 µm. The lack of fusion pores, on the other hand, are usually larger than the gas pores (100–150 µm), and they tend to take up a planar (perpendicular to building direction) and irregular shape within the layers of the produced parts [26,35,36]. The cubes produced from PPS 1 had some randomly distributed ovoid pores with diameters less than 90 µm, as can be seen in Figure 14b. The diameters of these randomly distributed pores were less than 100 µm; therefore, they can be considered to be gas pores, although they were not completely spherical [35]. The remainder of the pores were irregularly shaped, with diameters ranging from 100 to 245 µm, and are considered to be lack of fusion pores. Figure 14c displays the largest lack of fusion pore found in the cube. This pore had a diameter of 245 µm and exhibited small, rounded inclusions that formed on the surface of the cavity of the pore (yellow dotted circles). These inclusions resemble the morphology of the spherical elemental Ti powder, which was employed in the powder blend (Figure 1). It is thus safe to assume that this lack of fusion pore resulted from the lack of fusion of the powder blend constituents. 

The cube produced from PPS 2 (Figure 15) contained a mixture of gas pores and lack of fusion pores similar to those observed in the cube produced in PPS 1. According to the diameter scales (Figure 14a and Figure 15a), the sizes of the gas and lack of fusion pores in the cubes produced in PPS1 and PPS2 were in the same size range. The sizes of the gas pores produced with PPS 2 ranged from 30 to 90 µm, while the lack of fusion pores, on the other hand, had diameters ranging from 100 to 330 µm. However, despite the pores that were contained in the cubes, the overall density of the produced cubes was very satisfactory. The CT scanning results revealed that the cubes produced from PPS 1 and PPS 2 yielded total densities of 99.99%. These density values are higher than the in situ alloyed Ti6Al4V density values reported by other authors [11,12].

#### 3.2.5. Mechanical Properties

The mechanical properties obtained from the stress-relieved and annealed tensile test specimens and the properties specified in ASTM F2924-14 [16] are displayed in Table 3.

From Table 3, it is clear that the tensile specimens produced from the two process parameter sets yielded mechanical property values that were higher than the specified values for AM Ti6Al4V parts. A study conducted by Vrancken et al. [37] focused on the effect of different heat treatments on the microstructure and the resultant mechanical properties of DMLS-produced Ti6Al4V. In this study, Vrancken reported values of 948 ± 27 MPa, 899 ± 27 MPa and 13.59 ± 0.32% for the ultimate tensile strength (UTS), yield strength (Ys) and elongation at break (ε), respectively, for tensile specimens that were duplex annealed at 940 and 650 °C for 1 and 2 h, respectively. Becker et al. [31] reported UTS and ε values of 871 MPa and 11.5%, respectively, for Ti6Al4V specimens duplex annealed at 950 and 700 °C for 1 and 2 h, respectively. The values obtained in the current study are comparable with the values reported by Vrancken et al. and Becker et al. However, in this study, a single anneal HT was employed instead of a duplex anneal HT. Depending on the target application, a single anneal HT can be employed if a part with a high strength and adequate ductility is required. The duplex anneal HT, on the other hand, can be employed if a part that possesses a lower tensile strength and higher ductility is required [31,37]. The mechanical properties of the produced alloy are comparable to the mechanical properties of the wrought Ti6Al4V alloy presented in a review article by Atzeni et al. [38]. From the obtained results, it is evident that the pores that were present in the cubes displayed in Figure 14 and Figure 15 did not have a significant effect on the mechanical properties of the Ti6Al4V alloy produced from the two process parameter sets.

#### 3.2.6. Fractography

**Figure 16:**
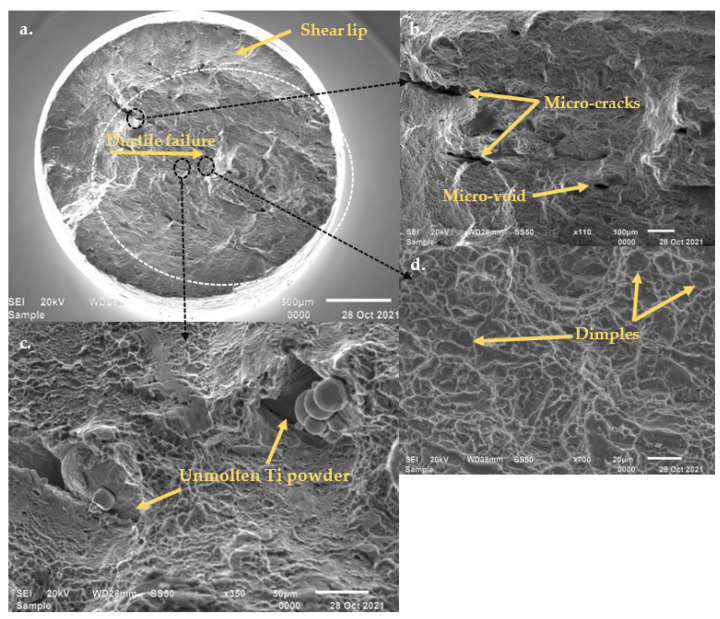
SEM SEI micrographs of the fracture surface of a tensile test specimen produced by PPS 1: (**a**) the overall fracture surface showing the shear lip and ductile failure in the central part of the fracture surface (crack initiation), (**b**) micro-cracks and voids, (**c**) unmolten Ti powder particles, (**d**) ductile region with dimples.

The fracture surfaces of the tensile specimens displayed a few areas with brittle features and some lack of fusion pores within an overall dominant ductile fracture surface, as can be seen in Figure 16. 

The overall fracture surface of the machined tensile specimen presented in Figure 16a displays the ductile crack initiation and shear lip (final fracture) areas of the fractured tensile specimen. The crack initiation zone appears to be circular and more fibrous as compared to the shear lip area, which is an indication that a slow transverse propagation of cracks occurred in this region. A shear lip area, which is inclined to the crack initiation and propagation plane, can be seen around the failure nucleation area, which indicates that pronounced necking occurred before the final, fast shear type fracture occurred. These features that were observed from the overall fracture surface are representative of a ductile type of fracture. A higher magnification of the fibrous failure nucleation area (Figure 16b) revealed a surface with randomly propagated micro-cracks and micro-voids (secondary cracks) that formed during the application of the tensile load. In Figure 16c, spherical unmolten Ti powders that resulted in a lack of fusion pore can be seen. This observation thus confirms the assumption that the small, rounded inclusions that were observed in Figure 16c were indeed unmolten spherical Ti powders, which resulted in a lack of fusion pore. These completely spherical, unmolten powders were observed throughout the range of optical analyses carried out in this study. Randomly scattered dimples within a ductile fibrous region can be seen in Figure 16d. These dimples are indicative of void nucleation and coalescence, which is common in ductile fracture [39].

The MA powder employed in this study was irregularly shaped and not spherical as is the requirement for powders employed in AM processes [40]. The individual powder particles within a feedstock interact and exert frictional forces onto each other; the morphology of the individual powders therefore directly affects the flowability and spreadability of the powder feedstock [41]. Spherical powders are therefore the preferred feedstock for AM processes due to their good flowability when they are delivered onto the powder bed by the recoater arm. Irregularly shaped powders within the feedstock negatively affect the flowability and spreadability of the powder, thereby resulting in the deposition of uneven layers during production and, ultimately, porosity in the produced components. Therefore, the recommendation is that a Ti-MA powder blend consisting of plasma-spheroidized MA powders be employed to improve the flowability and packing density of this powder blend.

## 4. Conclusions

The results obtained in this study demonstrate that in situ alloying can be employed to produce the Ti6Al4V alloy from a powder blend. The microstructures obtained before and after heat treatment were similar to the microstructures of parts that were produced from the PA Ti6Al4V powder. Consequently, the mechanical properties obtained from the tensile specimens were comparable to the mechanical properties that are typically associated with the dual phase microstructure obtained from heat treatment. Generally, the in situ alloyed Ti6Al4V parts were found to be of good-quality, high-density material.

From the obtained results, a laser power and scanning speed combination of 200 W; 1.2 m/s paired with a hatch distance of 80 µm using a layer thickness of 60 µm can be employed to produce a high-quality Ti6Al4V alloy. The Al evaporation observed from the alloy produced using these process parameters was 7.2%, and the final composition of the alloy was within the required limit for Ti6Al4V, as specified in the ASTM F2924-14 standard. However, the results obtained from this study can be improved by employing an Al–V MA powder, which contains Al and V compositions that are similar to the final required compositions. The employment of a powder blend consisting of completely spherical powders is a topic for further study, which could potentially increase the quality of the alloy.

## Figures and Tables

**Table 1:** Chemical composition of the powders employed in the Ti-MA powder blend, as determined by ICP-OES.

Powder Material	Ti	Al	V	Fe	O	N
CP Ti	99.3	0.1	0.1	0.12	0.17	0.31
Al–V MA	0.1	54.8	43.4	0.64	0.18	0.88

**Table 2:** ICP-OES composition determined for cubes produced with PPS 1 and PPS 2 as compared with the ASTM F2924-14 specification.

Element	PPS 1	PPS 2	ASTM F2924–14
Ti	91.4	90.8	Remainder
Al	4.73	5.11	5.5–6.75 ± 0.4
V	3.53	3.72	3.5–4.5 ± 0.15
O	0.19	0.21	0.2 ± 0.02
N	0.00433	0.00619	0.05 ± 0.02
Fe	0.07	0.05	0.3 ± 0.1

**Table 3:** The mechanical properties obtained from the heat-treated Ti-MA tensile specimens compared to the ASTM F2924–14 standard.

Property	Process Parameter Set 1	Process Parameter Set 2	ASTM F2924–14
UTS (Mpa)	932.3	942.9	895
Ys (Mpa)	932.2	942.9	825
ε (%)	12.9	17	10
Area reduction (%)	30.6	35.9	15

## Data Availability

Not applicable.
